# Supplementary figures and images for: A phenotype-based forward genetic screen identifies Dnajb6 as a sick sinus syndrome gene
Source: eLife. 2022 Oct 18;11:e77327. doi: 10.7554/eLife.77327 (PMC9642998; doi:10.7554/eLife.77327)

## Slide 1
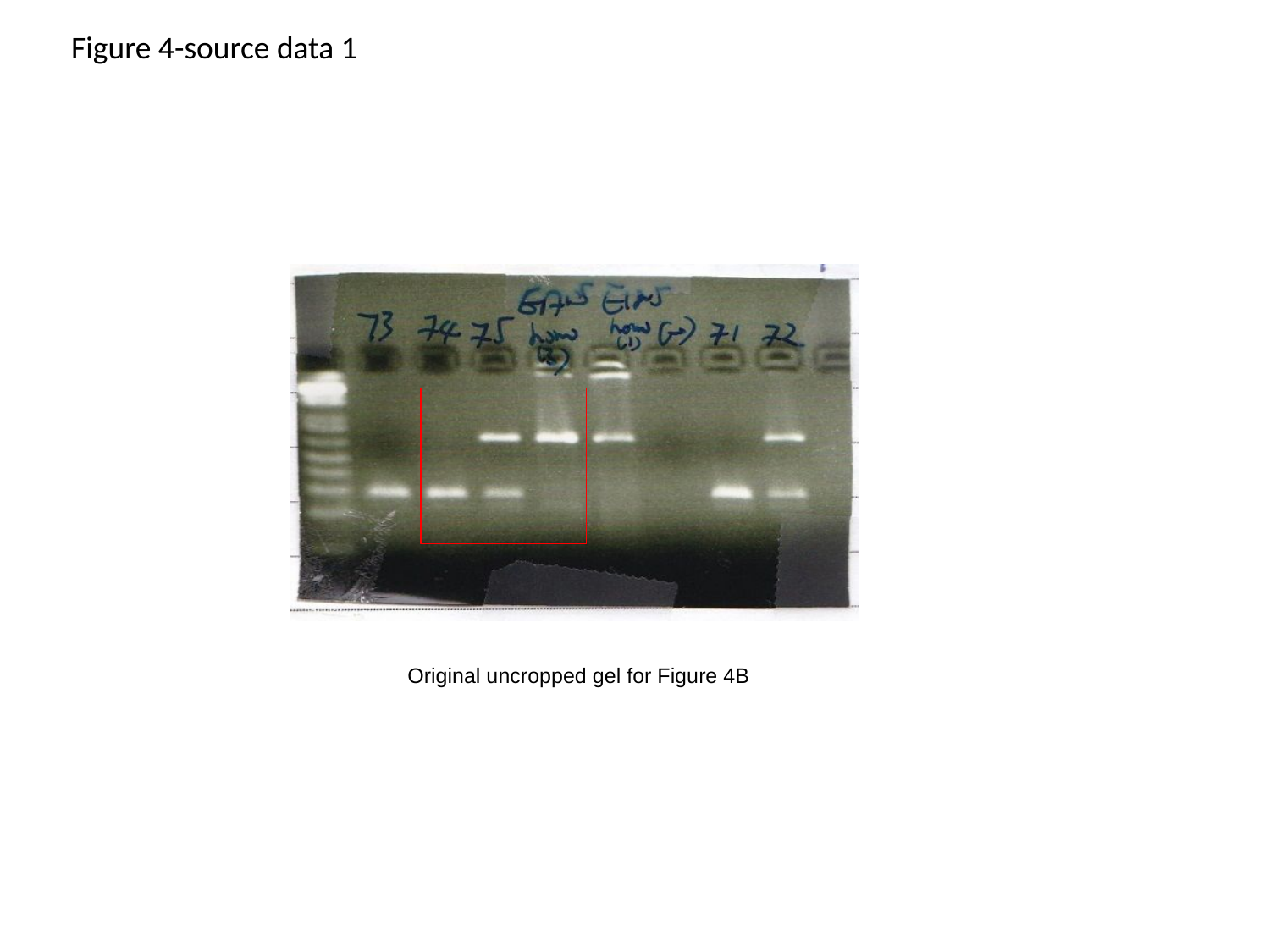

Figure 4-source data 1
Original uncropped gel for Figure 4B

Supplement: Figure 4—source data 1. [file elife-77327-fig4-data1.zip › Figure 4source data 1.pptx]

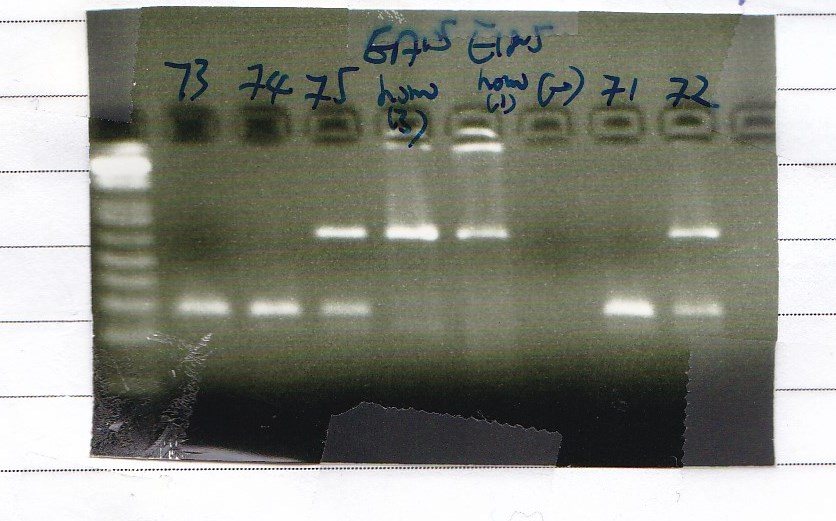

Supplement: Figure 4—source data 2. [file elife-77327-fig4-data2.zip › Xu_24-01-2022-ADV-eLife-77327R1_Figure_4_source_data_2.jpg]

## Slide 1
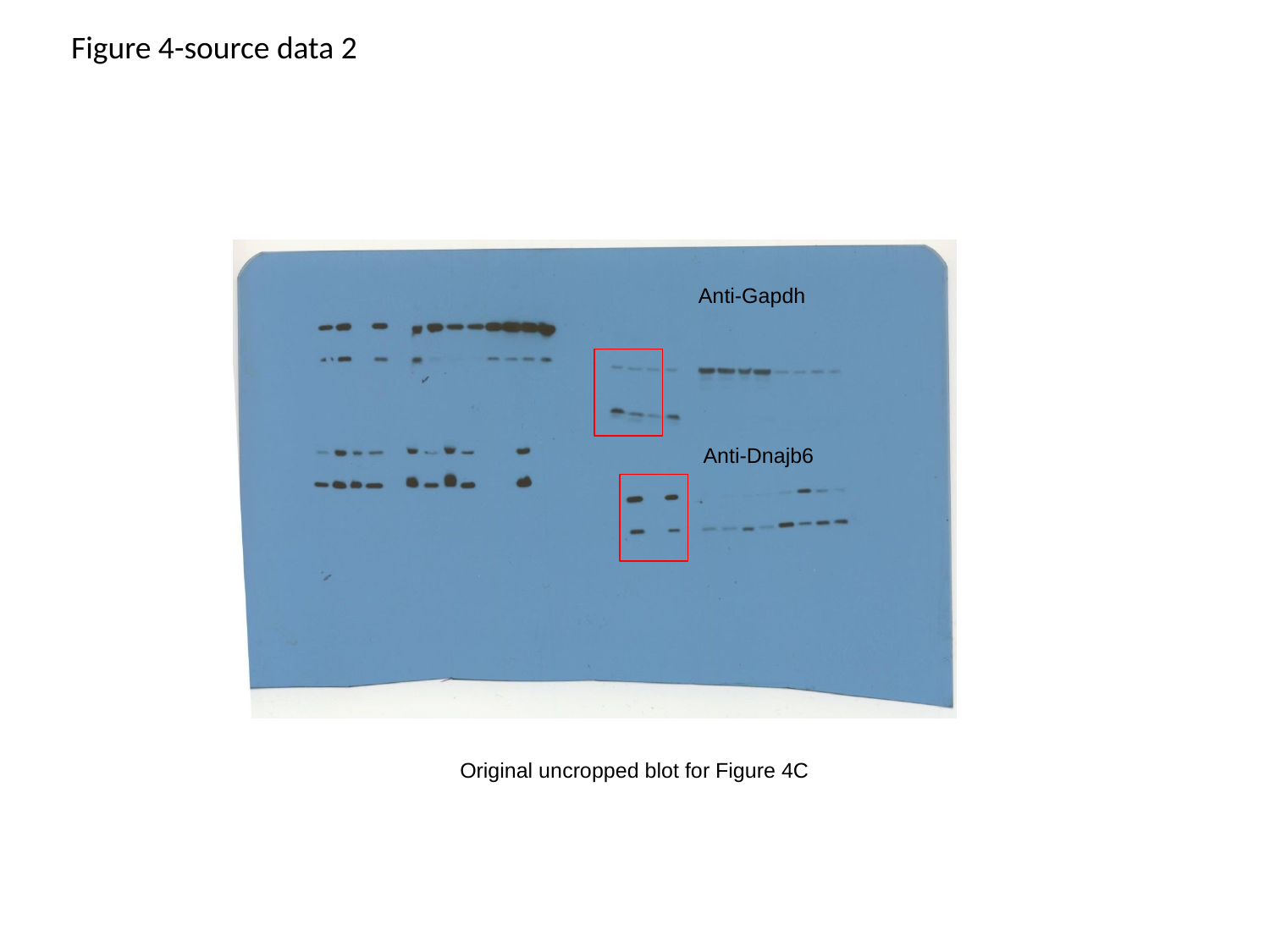

Figure 4-source data 2
Anti-Gapdh
Anti-Dnajb6
Original uncropped blot for Figure 4C

Supplement: Figure 4—source data 3. [file elife-77327-fig4-data3.zip › Figure 4source data 2.pptx]

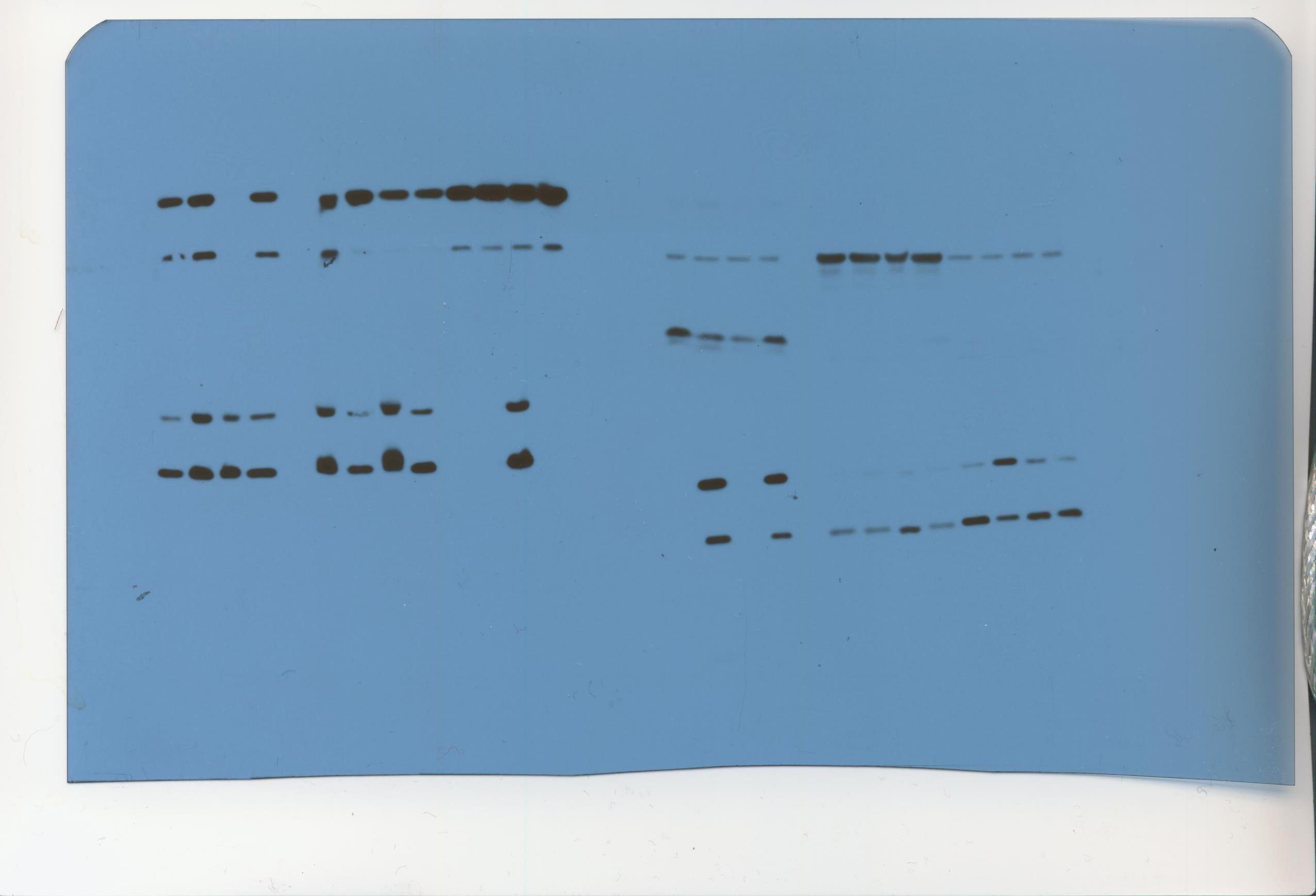

Supplement: Figure 4—source data 4. [file elife-77327-fig4-data4.zip › Xu_24-01-2022-ADV-eLife-77327R1_Figure_4_source_data_4.jpg]
