# Supplementary material for: A phenotype-based forward genetic screen identifies Dnajb6 as a sick sinus syndrome gene
Source: eLife. 2022 Oct 18;11:e77327. doi: 10.7554/eLife.77327 (PMC9642998; doi:10.7554/eLife.77327)
Supplement: Supplementary file 1. — SA, sinus arrest. epm, episode per minute. bpm, beats per minute. N=8–9. *, P<0.05, data are expressed as mean ± SEM. For SA incidence comparison, Chi-square test. For heart rate comparison, unpaired student t-test. [file elife-77327-supp1.docx]

**Supplementary File 1.** ECG quantification of *GBT411^-/-^* mutant at 10 months of age

|  | **Genotype** | **Age** | **N** | **SA incidence (%)** | **SA Frequency (epm)** | **Heart rate (bpm)** |
| --- | --- | --- | --- | --- | --- | --- |
| Baseline | WT | 10 m | 8 | 0(0.0) | - | 108.8±25.5 |
|  | *GBT411^-/-^* | 10 m | 8 | 9(12.5) | 1.0 | 102.6±9.3 |
| Verapamil | WT | 10 m | 8 | 2(25.0) | 3.4±0.6 | 80.3±11.5 |
|  | *GBT411^-/-^* | 10 m | 9 | 8(88.9)* | 5.2±3.2 | 71.5±8.8* |

SA, sinus arrest. epm, episode per minute. bpm, beats per minute. N=8-9. *, *P*<0.05, data are expressed as mean±SEM. For SA incidence comparison, Chi-square test. For heart rate comparison, unpaired student *t-*test.
