# Supplementary material for: A phenotype-based forward genetic screen identifies Dnajb6 as a sick sinus syndrome gene
Source: eLife. 2022 Oct 18;11:e77327. doi: 10.7554/eLife.77327 (PMC9642998; doi:10.7554/eLife.77327)
Supplement: Supplementary file 2. — N=8. *, P<0.05, data are expressed as mean ± SEM. [file elife-77327-supp2.docx]

**Supplementary File 2.** ECG quantification of *GBT411^-/-^* mutant at 16 months of age

| **Genotype** | **Age** | **N** | **Heart rate (bpm)** | **PR interval**  **(ms)** | **QRS duration**  **(ms)** | **QT interval (ms)** | **RR interval (ms)** |
| --- | --- | --- | --- | --- | --- | --- | --- |
| WT | 16 m | 8 | 103.0±10.4 | 100.0±9.3 | 111.3±12.5 | 345.0±51.3 | 587.9±61.3 |
| *GBT411^-/-^* | 16 m | 8 | 90.3±9.5* | 106.3±9.2 | 97.5±14.9 | 346.3±97.5 | 670.9±66.4* |

N=8. *, *P*<0.05, data are expressed as mean±SEM.
