# Supplementary material for: A phenotype-based forward genetic screen identifies Dnajb6 as a sick sinus syndrome gene
Source: eLife. 2022 Oct 18;11:e77327. doi: 10.7554/eLife.77327 (PMC9642998; doi:10.7554/eLife.77327)
Supplement: Supplementary file 3. — bpm, beats per minute. N=8 *, P<0.05, unpaired student’s t-test. [file elife-77327-supp3.docx]

**Supplementary File 3.** ECG quantification of *Dnajb6^+/-^* mice at 6 months of age

| **Genotype** | **Age** | **N** | **Heart rate (bmp)** | **PR interval (ms)** | **QRS duration (ms)** | **QT interval (ms)** | **RR interval (ms)** |
| --- | --- | --- | --- | --- | --- | --- | --- |
| WT | 6 m | 8 | 518.4±12.5 | 53.6±5.2 | 13.3±1.4 | 48.4±8.4 | 116.2±6.3 |
| *Dnajb6+/-* | 6 m | 8 | 495.5±17.0* | 51.1±9.4 | 14.4±1.5 | 52.8±5.8 | 121.3±7.4* |

bpm, beats per minute. N=8 *, *P*<0.05, unpaired student’s *t-*test.
