# Supplementary material for: A phenotype-based forward genetic screen identifies Dnajb6 as a sick sinus syndrome gene
Source: eLife. 2022 Oct 18;11:e77327. doi: 10.7554/eLife.77327 (PMC9642998; doi:10.7554/eLife.77327)
Supplement: Supplementary file 4. — aThe effect that the variant has on each feature that it overlaps. bA subjective classification of the severity of the variant consequence, based on agreement with SNPEff. SNP, single nucleotide polymorphism; MAF minor allele frequency; A1, minor allele; A2, major; OR, odds ratio; SE, standard error; info, information metric (info score). [file elife-77327-supp4.docx]

**Supplementary File 4.** Variants in *DNAJB6* associated with sick sinus syndrome with *P*<0.05

| **SNP** | **MAF** | **A1** | **A2** | ***P* value** | **OR** | **SE** | **Info** | **Consequence^a^** | **IMPACT^b^** |
| --- | --- | --- | --- | --- | --- | --- | --- | --- | --- |
| ***rs754941044*** | 0.011 | G | GGAGAAAGGA | 0.0193 | 0.728 | 0.136 | 0.993 | splice_acceptor_variant,splice_polypyrimidine_tract_variant,5_prime_UTR_variant,intron_variant | HIGH |
| ***rs35152520*** | 0.027 | G | GGT | 0.0398 | 0.856 | 0.076 | 0.994 | 3_prime_UTR_variant | MODIFIER |
| ***rs117451998*** | 0.028 | A | G | 0.0448 | 0.858 | 0.077 | 0.992 | intron_variant,NMD_transcript_variant | MODIFIER |
| ***rs6974584*** | 0.442 | T | C | 0.0469 | 0.961 | 0.020 | 0.995 | intron_variant,NMD_transcript_variant | MODIFIER |

^a^The effect that the variant has on each feature that it overlaps. ^b^A subjective classification of the severity of the variant consequence, based on agreement with SNPEff. SNP, single nucleotide polymorphism; MAF minor allele frequency; A1, minor allele; A2, major; OR, odds ratio; SE, standard error; info, information metric (info score).
