# Supplementary material for: A phenotype-based forward genetic screen identifies Dnajb6 as a sick sinus syndrome gene
Source: eLife. 2022 Oct 18;11:e77327. doi: 10.7554/eLife.77327 (PMC9642998; doi:10.7554/eLife.77327)
Supplement: Supplementary file 5. [file elife-77327-supp5.docx]

**Supplementary File 5.** Quantitative RT-PCR primers used to validate differentially expressed (DE) genes identified from RNAseq

| Primer name | Primer Sequence |
| --- | --- |
| Gapdh-F | 5’-AGGTCGGTGTGAACGGATTTG-3’ |
| Gapdh-R | 5’- GTAGACCATGTAGTTGAGGTCA-3’ |
| Slc24-F | 5’- AGCAACAGAGGAGAAGCCAG-3’ |
| Slc24-R | 5’- TCACAGACGATGGCTAATGC-3’ |
| Dixdc1-F | 5’- CTGCAAGAGGGCTTCAATG-3’ |
| Dixdc1-R | 5’- AACTGACCAACAATCTCGATG-3’ |
| Slc9-F | 5’- GTGAACGGTGAGAATGTGG-3’ |
| Slc9-R | 5’- TTTCTTCATGGTGCAGAGC-3’ |
| Gjb-F | 5’- GAAGGTTACCTTTACCCGAATC-3’ |
| Gjb-R | 5’- GGTTAAGCAGGATGCAGATG-3’ |
| Wnt2-F | 5’- CTCGGTGGAATCTGGCTCTG-3’ |
| Wnt2-R | 5’- CACATTGTCACACATCACCCT-3’ |
| Wnt9b-F | 5’- AAGAGAGGAAGCAAGGACC-3’ |
| Wnt9b-R | 5’- TCCAACAGGTACGAACAGC-3’ |
| Ccnd1-F | 5’- GTGAAGTTCATTTCCAACCCAC-3’ |
| Ccnd1-R | 5’- TGACCGGGTCACACTTGATG-3’ |
| FXYD5-F | 5’- CCAAACCGAGACCCAGCAA-3’ |
| FXYD5-R | 5’- AACTGCCTACACTTCCCACTA-3’ |
| Kcnh7-F | 5’- CCAGGAAACTGGACCGATACT-3’ |
| Kcnh7-R | 5’- CCAATCGCATACCAGATGCAA-3’ |
| Cdh20-F | 5’- ATGTGGACTACGGGTAGAATGA-3’ |
| Cdh20-R | 5’- GTGCTGGAGAGAACAGTGGC-3’ |
