# Supplementary material for: A phenotype-based forward genetic screen identifies Dnajb6 as a sick sinus syndrome gene
Source: eLife. 2022 Oct 18;11:e77327. doi: 10.7554/eLife.77327 (PMC9642998; doi:10.7554/eLife.77327)
Supplement: Supplementary file 6. [file elife-77327-supp6.docx]

**Supplementary File 6.** Definition of model parameters and changes induced by isoproterenol (ISO) and carbachol (CCh) administration.

| ***Ion channels*** | | |
| --- | --- | --- |
| **Maximal conductances** | | **ISO- or CCh-dependent effects** |
| G_st_ | Sustained inward Na^+^ current (I_st_) | ISO: 15% increase in maximal conductance  (Kharche et al. 2011) |
| G_Na1.1_ | TTX-sensitive Na^+^ current (I_Na1.1_) |  |
| G_Na1.5_ | TTX-resistant Na^+^ current (I_Na1.5_) | ISO: 15% increase in maximal conductance  (Kharche et al. 2011) |
| G_CaT_ | T-type Ca^2+^ current (I_CaT_) | ISO: 75% increase in maximal conductance  (Larson et al. 2013) |
| G_CaL_ | L-type Ca^2+^ current (I_CaL_) | ISO: 2-fold increase in maximal conductance  (Larson et al. 2013) |
| G_f_ | Hyperpolarization-activated (funny) current (I_f_) | ISO: gain of function via altered steady-state activation and gating properties  (Peters et al. 2021) |
| G_K1_ | Time-independent K^+^ current (I_K1_) | ISO: 15% decrease in maximal conductance (Kharche et al. 2011) |
| G_Kr_ | Rapid delayed rectifying K^+^ current (I_Kr_) | ISO: 10% increase in maximal conductance and 5-mV shift of steady-state activation to  more negative potentials (Kharche et al. 2011) |
| G_Ks_ | Slow delayed rectifying K^+^ current (I_Ks_) | ISO: 15% increase in maximal conductance  (Kharche et al. 2011) |
| G_to_ | Transient component of the 4-AP-sensitive K^+^ current (I_to_) | ISO: 15% increase in maximal conductance  (Kharche et al. 2011) |
| G_sus_ | Sustained component of the 4-AP-sensitive K^+^ current (I_sus_) |  |
| G_NaB_ | Background Na^+^ current (I_NaB_) |  |
| G_CaB_ | Background Ca^2+^ current (I_CaB_) |  |
| G_K,ACh_ | Acetylcholine-dependent K^+^ current (I_K,ACh_) | CCh: ion current activation as in Arbel-Ganon et al. (2020) |
| ***Ion transporters*** | | |
| **Maximal transport rates** | | **ISO- or CCh-dependent effects** |
| v_NKA_ | Na^+^/K^+^ ATPase (NKA) |  |
| v_NCX_ | Na^+^/Ca^2+^ exchanger (NCX) |  |
| v_RyR_ | Ca^2+^ release via ryanodine receptor (RyR) | ISO: enhanced Ca^2+^ release via 2-fold increase in KoCa (non-SR-dependent transition rate constant) (Kharche et al. 2011) |
| v_SERCA_ | Sarcoplasmic reticulum (SR) Ca^2+^ pump (SERCA) | ISO: enhanced Ca^2+^ uptake via 50% decrease in Kmf to reduce Ca^2+^-affinity (Kharche et al. 2011) |
